# Knowledge, Awareness, and Attitudes Regarding Self-Medication With Non-opioid Analgesics Among Students in Hafar Al-Batin, Saudi Arabia: A Cross-Sectional Study

**DOI:** 10.7759/cureus.73512

**Published:** 2024-11-12

**Authors:** Emad Hamdy Mohammad Ismail, Abdullah K Dabaan, Ghaday Almutairi, Fatmah A Alhumidan, Mariam Abdulrhaman Palani Sami, Ghada M Aldhafeeri, Farah Nasser Hamdan Alsuwayt, Muzun Salahaldeen Rhamatalla Fadul, Mohammed S Alotaibi, Moayad A AlFurayh, Abdullah S Alotaibi, Abdalrhman Raed Deab Aesa

**Affiliations:** 1 Emergency Department, King Khalid General Hospital, Hafar Al-Batin, SAU; 2 College of Medicine, Qassim University, Buraydah, SAU; 3 College of Medicine, Arabian Gulf University, Manama, BHR; 4 College of Medicine, Sulaiman Al Rajhi University, Al Bukayriyah, SAU; 5 College of Medicine, Hail University, Hail, SAU; 6 College of Medicine, Ibn Sina University, Khartoum, SDN; 7 College of Medicine, Alexandria University, Alexandria, EGY; 8 College of Medicine, Taif University, Ta'if, SAU

**Keywords:** attitude, knowledge, non-opioid analgesics, saudi arabia, self-medication

## Abstract

Introduction: Self-medication (SM) with non-opioid analgesics (NOAs) has become increasingly prevalent, with individuals using over-the-counter medications to manage pain and other symptoms without professional guidance. While NOAs are generally considered safe when used appropriately, misuse or overuse can lead to adverse effects, including gastrointestinal issues, liver damage, and renal problems.

Aim: This study aimed to evaluate the knowledge, awareness, and attitudes regarding NOAs and their SM practices, including how these factors vary by demographic characteristics.

Subject and methods: This cross-sectional study utilized data from a sample of 475 students who were randomly selected among university students at the University of Hafr Albatin, Hafar Al-Batin, Saudi Arabia. The selected students completed the validated self-administered online questionnaires. The questionnaire includes socio-demographic characteristics (e.g., age, gender, marital status, etc.), the use of SM with analgesics, and 20 items to measure students' knowledge, awareness, and attitude regarding NOAs for SM.

Results: All the students sampled responded to our survey representing a 100% response rate. Of the participants, more than half 296 (62.3%) were females, and about 289 (60.8%) were in the 18-to-21 years old age group. The prevalence of students who were using SM with analgesics was about 61.3% (N=291). More than half of the students (276, 58.1%) were regarded as having good knowledge about NOAs for SM; however, only 186 (39.2%) were considered to have a positive attitude about it. Parents' higher education, having health insurance, and current use of SM with analgesics were associated with increased knowledge and attitude. Interestingly, we noted a significant positive correlation between knowledge and attitude scores (p<0.001).

Conclusion: Despite students showing favorable knowledge of NOAs for SM, their attitude about it was less than desired. Students’ knowledge and attitude regarding NOAs for SM increased significantly depending on their parents' education, monthly income, and health insurance. Further, this study highlights that students' knowledge was positively correlated with their attitude regarding non-opioid SM. Prospective studies are needed to determine the cause and effect of these factors.

## Introduction

According to the World Health Organization (WHO) in 2015, self-medication (SM) refers to the use of medicinal products by individuals to manage self-recognized disorders or symptoms or the continued use of a physician-prescribed medication for chronic or recurring conditions [1]. The significance of SM in public health has grown, especially with the shift of some medications to over-the-counter (OTC) status, allowing them to be purchased without a prescription. This change aims to alleviate the workload on healthcare professionals. However, the availability of analgesic medications varies widely across different countries [2].

SM with non-opioid analgesics (NOAs) has become increasingly prevalent, with individuals using OTC medications to manage pain and other symptoms without professional guidance. While NOAs are generally considered safe when used appropriately, misuse or overuse can lead to adverse effects, including gastrointestinal issues, liver damage, and renal problems. Studies have shown that public knowledge and attitudes regarding SM can significantly impact the safe use of these medications [3,4]. In the Eastern Province of Saudi Arabia, there is a growing concern about the patterns of SM and the level of public awareness regarding the risks and proper use of NOAs.

SM involves using medications to treat self-diagnosed conditions without professional medical advice [5]. This can include OTC drugs, prescription medications, pharmacist-only medicines, complementary and alternative therapies [5,6], following recommendations from relatives, or using leftover medicines from home [7]. SM is a common practice in both developing and developed countries as part of self-care [8]. When done appropriately, SM offers several benefits, such as preventing and treating minor illnesses and providing cost-effective alternatives [9]. However, irrational SM characterized by misdiagnosis, incorrect medication selection, misunderstanding of drug labels and leaflets, and unawareness of drug interactions can lead to ineffective treatment, drug resistance, serious side effects, interactions with food and other drugs, allergies, delays in proper diagnosis, overdose, and toxicity [10].

SM, particularly with NOAs, has emerged as a global public health concern due to its widespread practice and associated risks. The convenience of OTC medications contributes to high rates of SM, but this often occurs without appropriate medical guidance, leading to potential health risks.

SM with analgesics is prevalent across various regions. Studies indicate high rates of SM among university students globally, with significant variations in prevalence. For instance, in Bangladesh, South India, and Belgrade, prevalence rates of SM among medical students were reported at 88.0%, 87.3%, and 79.9%, respectively. Analgesics usage among these students was at 49.2%, 72.5%, and 55.4% [11-13].

In the Middle East, including Egypt, the accessibility of OTC medications exacerbates the issue of SM. Over the past two decades, Egypt has experienced a notable rise in medication abuse, with a prevalence rate of 86.4%. Analgesics are the most commonly abused drugs, representing 96.7% of cases [14]. A study in Minia, Egypt, found that 73% of 422 randomly selected adults engaged in SM [15].

SM is widespread in developing countries, with reports indicating that prescription medications can often be obtained without a prescription [16]. In Saudi Arabia, as in many other countries, private-sector pharmacies are the most accessible healthcare providers. The prevalence of SM in Arab countries, including Saudi Arabia, ranges from 35% to 92% [17]. A study conducted by Aljadhey et al. [18] in Saudi Arabia revealed high rates of SM among residents. Notably, 22% reported using antibiotics without a prescription, while 68.6% relied on advice from family and friends for SM. Additionally, 50.4% engaged in self-treatment for oral health issues. These findings highlight both the prevalence of SM practices and the potential risks associated with inappropriate use.

The prevalence of SM among various populations raises significant public health concerns including inaccurate diagnosis, misuse of medication, and delayed treatment, which potentially worsen health outcomes. The reviewed studies suggest several key interventions: targeted educational programs are essential to mitigate the risks associated with SM, and enhanced regulations on OTC medications may help curb inappropriate use. Strengthening the role of pharmacists in providing accurate drug information is also crucial, as is increasing public awareness about the dangers of SM and encouraging consultations with healthcare professionals. Collectively, these measures aim to address the challenges posed by SM and promote safer practices.

Given the widespread use and potential risks of these medications, understanding public perceptions and practices is crucial for promoting safe usage. This study aims to evaluate the knowledge, awareness, and attitudes of students in Hafar Al-Batin, Saudi Arabia, regarding SM with NOAs. Furthermore, analyzing the impact of demographic factors will help tailor interventions to students' characteristics, enhancing the overall effectiveness of health strategies in the region.

## Materials and methods

This cross-sectional study was conducted among the university students of the Faculty of Nursing, Pharmacy, and Applied Medical Sciences who were aged 18 years and above at Hafar Al-Batin University. The study employed a random sampling technique. The Eastern Province of Saudi Arabia has a population of approximately 376000, as reported by the Saudi Census Department [19], while the total number of students in Hafar Al-Batin University was 23,785 in 2023, according to the official university website [20]. To determine the required sample size for our study, the Raosoft sample size calculator has been used with the formula $N = \frac{Z^2 \cdot p \cdot (1 - p)}{E^2}$, where (N) is the population size, (Z) is the critical value corresponding to the desired confidence level. With a 5% margin of error and a 95% confidence level, the minimum sample size calculated was 385, we increased 25% of the calculated sample to cover the non-response rate. The total sample population being collected was 475.

Participants were primarily recruited through convenience sampling by visiting residents' halls, libraries, and lecture halls. Additionally, the validated questionnaire was disseminated via various social media platforms in Hafar Al-Batin, Saudi Arabia, including Twitter, Facebook, LinkedIn, Instagram, and WhatsApp Status. We employed a snowball sampling technique through personal networks to further enhance the number of responses. Adult students aged 18 years or older who lived in the Eastern Province of Saudi Arabia were included, while individuals less than 18 years, non-Eastern Region residents, and those who did not use non-opioid medications were excluded.

Data were collected using a self-administered, structured questionnaire in Arabic. The questionnaire was divided into two main sections: the first section gathered demographic information about the adult participants, while the second section focused on their SM practices. The study was conducted following ethical guidelines and approved by a relevant ethics committee. Written informed consent was obtained from all participants, and their responses were kept anonymous and confidential.

Questionnaire criteria

The knowledge of NOAs has been assessed using a 10-item validated questionnaire, with the correct answer being identified and coded with 1, while the incorrect answer being coded with 0. The total knowledge score has been calculated by adding all 10 items. Scores ranging from 0 to 10 points have been obtained. The higher the score, the higher the knowledge of NOAs. By using 50% and 75% as cutoff points to determine the level of knowledge, students were considered to have poor knowledge if the score was below 50%, 50% to 75% were moderate, and above 75% were considered to have good knowledge levels.

Likewise, the attitude regarding NOAs has been assessed using a 10-item questionnaire, with a 3-point Likert scale category ranging from "disagree" coded with 1 to "agree" coded with 3 as the answer options. By summing up the 10 items, a score ranging from 3 to 30 points has been achieved. The greater the score, the more positive attitude regarding NOAs. Similar thresholds were applied to determine the level of attitude, such as negative (<50%), neutral (50-75%), and positive attitude (>75%).

Statistical analysis

Categorical variables were shown as numbers and percentages, while continuous variables were calculated and presented as mean and standard deviation. The differences in the knowledge and attitude scores in relation to students' socio-demographic characteristics have been conducted using the Mann-Whitney Z-test test. The normality test was performed using the Kolmogorov-Smirnov test. The knowledge and attitude scores follow the non-normal distribution. Thus, the non-parametric test was applied. Statistical significance has been set to p<0.05 level. All statistical data were analyzed using Statistical Package for the Social Sciences (IBM SPSS Statistics for Windows, IBM Corp., Version 26.0, Armonk, NY).

## Results

A total of 475 university students responded to our survey. Table 1 presents the socio-demographic characteristics of university students. The most common age group was 18 to 21 years (N=289; 60.8%), with females being prevalent (N=296; 62.3%). Nearly all were single (N=456; 96%). The most common specialty was pharmacy (N=212; 44.6%). Regarding the academic year level, 133 (28%) were in the second-year level. A vast majority of the students lived with their families (N=412; 86.7%). With respect to the parent's education, 279 (58.7%) (father) and 252 (53.1%) (mother) had university or higher degrees. Approximately 361 (76%) indicated having a sufficient monthly income. The proportion of students who had health insurance was 275 (57.9%). In addition, the prevalence of students who were using SM with analgesics was 291 (61.3%).

**Table 1:** Socio-demographic characteristics of the university students (N=475)

Demographic variables	N (%)
Age group	
18-21 years	289 (60.8)
22-25 years	167 (35.2)
>25 years	19 (4.0)
Gender	
Male	179 (37.7)
Female	296 (62.3)
Marital status	
Single	456 (96.0)
Married	19 (4.0)
Specialty	
Pharmacy	212 (44.6)
Nursing	106 (22.3)
Health administration	42 (8.8)
Nutrition	29 (6.1)
Preparing for a healthy path	35 (7.4)
Medical laboratory	51 (10.7)
Academic year	
First year	102 (21.5)
Second year	133 (28.0)
Third year	106 (22.3)
Fourth year	53 (11.2)
Fifth year	36 (7.6)
Sixth year	20 (4.2)
Internship	25 (5.3)
Living status	
Living with family	412 (86.7)
Living with dormitory	63 (13.3)
Father education	
Less than university	196 (41.3)
University or higher	279 (58.7)
Mother education	
Less than university	223 (46.9)
University or higher	252 (53.1)
Average monthly income	
Insufficient	114 (24.0)
Sufficient	361 (76.0)
Health insurance	
No	200 (42.1)
Yes	275 (57.9)
Use of self-medication with analgesics	
No	184 (38.7)
Yes	291 (61.3)

**Figure 1:**
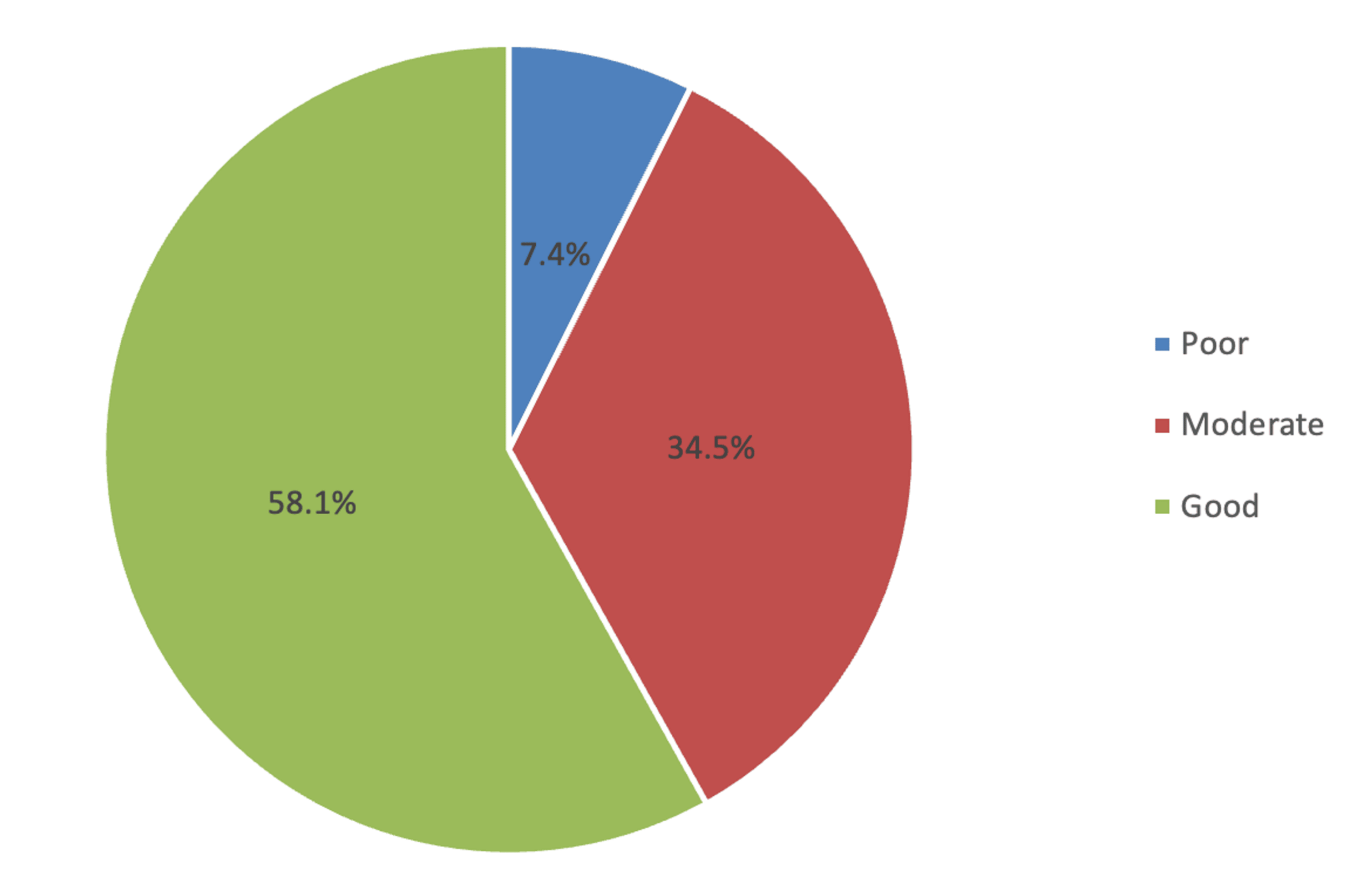
Level of knowledge regarding non-opioid analgesics self-medication

**Figure 2:**
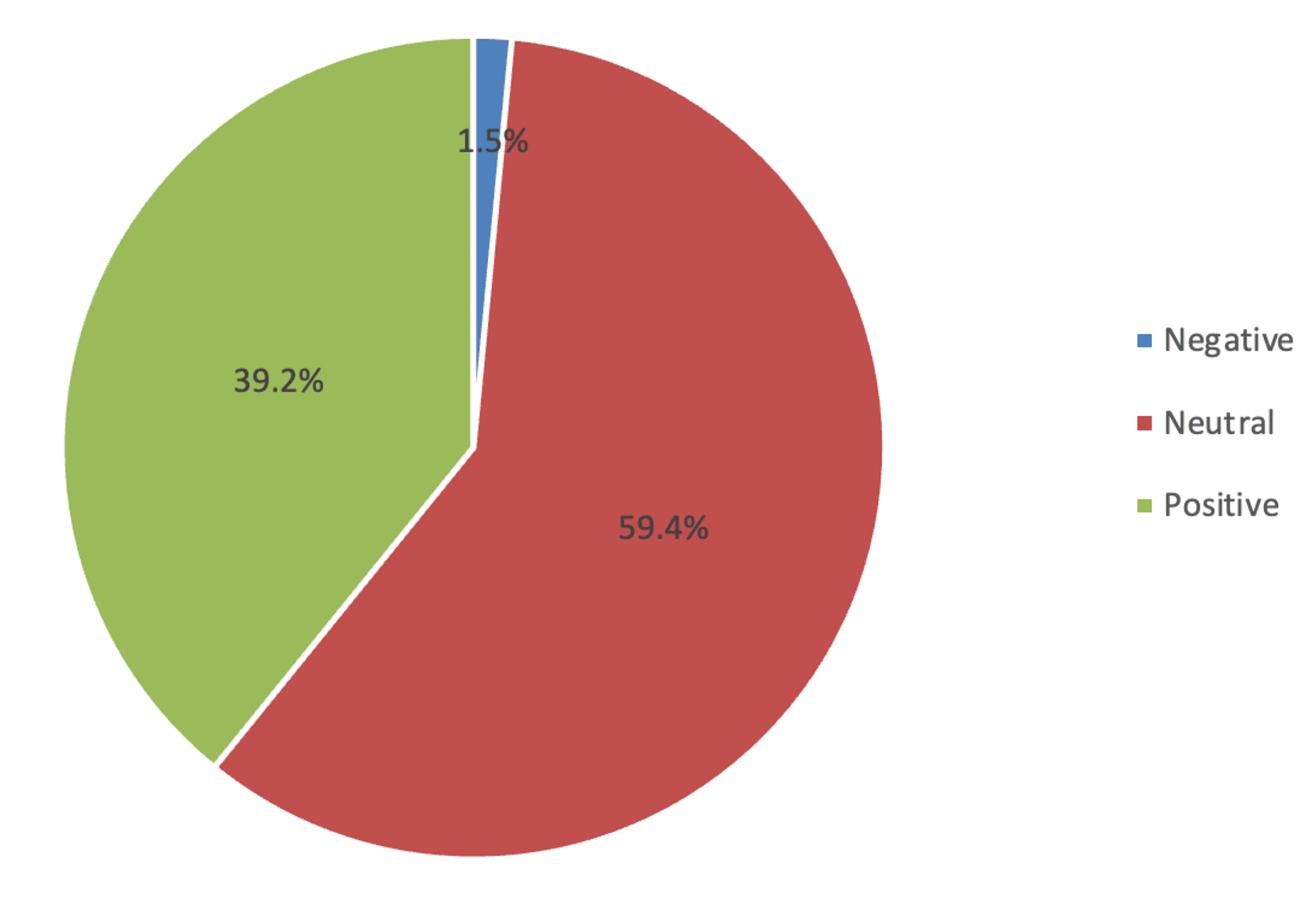
Level of attitude regarding non-opioid analgesics self-medication

Regarding the knowledge about NOAs SM (Table 2), it was observed that most students were knowledgeable in all knowledge items as they were able to identify the correct answer for each knowledge item, with the highest ratings seen in the correct definition of analgesic drugs (N=446; 93.9%), followed by the drug that could cause liver toxicity in large doses (N=411; 86.5%) and analgesic drug that has a weak anti-inflammatory activity (N=407; 85.7%). The lowest rating was seen in the items, analgesic drugs that produce an anticoagulant effect with a prolonged bleeding time (N=375; 78.9%) and an analgesic drug, which is not among the non-steroidal anti-inflammatory drugs (NSAIDs) (N=375; 78.9%). The overall mean knowledge score was 8.44 (standard deviation, or SD 1.84), with poor, moderate, and good knowledge constituting 35 (7.4%), 164 (34.5%), and 276 (58.1%), respectively (Figure 1). Regarding the attitude regarding non-opioid SM, the top three statements with the highest ratings were "I think that self-medication with analgesics may cause interaction with other drugs" (mean score: 2.73), followed by "I think that self-medication with analgesics may have a negative impact" (mean score: 270), and "I think that self-medication with analgesics may cause side effects" (mean score: 2.70), while statement "I think that I can use analgesics irrespective of the seriousness of the illness" (mean score: 1.81) showed the lowest rating. The overall mean attitude score was 23.2 (SD 4.08). Accordingly, seven (1.5%), 282 (59.4%), and 186 (39.2%) were classified as negative, neutral, and positive attitude levels (Figure 2).

**Table 2:** Assessment of knowledge and attitude regarding non-opioid analgesics self-medication (N=475) Attitude item has category responses of "disagree" coded with 1, "neutral" coded with 2, and "agree" coded with 3. NSAIDs: non-steroidal anti-inflammatory drugs

Knowledge items	N (%)
The correct definition of analgesic drugs (Analgesics are medications used to relieve pain without causing loss of consciousness)	446 (93.9)
A drug which could cause liver toxicity in large doses (acetaminophen)	411 (86.5)
Analgesic drug that has a weak anti-inflammatory activity (acetaminophen)	407 (85.7)
Contraindication and side effects of aspirin (peptic ulcer)	406 (85.5)
The safest analgesic during pregnancy (paracetamol)	406 (85.5)
Type of diclofenac drug (NSAIDs)	398 (83.8)
A drug that has anti-inflammatory, antipyretic, and analgesic effects (aspirin)	394 (82.9)
The best analgesic to treat a child who has a viral infection (acetaminophen)	393 (82.7)
Analgesic drug which is not among the NSAIDs (paracetamol)	375 (78.9)
Analgesic drugs that produce an anticoagulant effect with a prolonged bleeding time (NSAIDs)	375 (78.9)
Total knowledge score (mean ± SD)	8.44 ± 1.84
Level of knowledge	
Poor	35 (07.4)
Moderate	164 (34.5)
Good	276 (58.1)
Attitude items	Mean ± SD
I think that self-medication with analgesics may cause interaction with other drugs	2.73 ± 0.53
I think that self-medication with analgesics may have a negative impact	2.70 ± 0.55
I think that self-medication with analgesics may cause side effects	2.70 ± 0.54
I think that self-medication enhances the inappropriate use of analgesics	2.55 ± 0.65
I think that medical students can diagnose diseases	2.36 ± 0.73
I'm satisfied with self-medication as a method of treatment	2.26 ± 0.75
I think that self-medication with analgesics saves time and money	2.07 ± 0.85
I think that medical students can treat themselves	2.13 ± 0.78
I think that I can share analgesics with relatives	1.93 ± 0.86
I think that I can use analgesics irrespective of the seriousness of the illness	1.81 ± 0.88
Total attitude score	23.2 ± 4.08
Level of attitude	N (%)
Negative	07 (01.5)
Neutral	282 (59.4)
Positive	186 (39.2)

**Figure 3:**
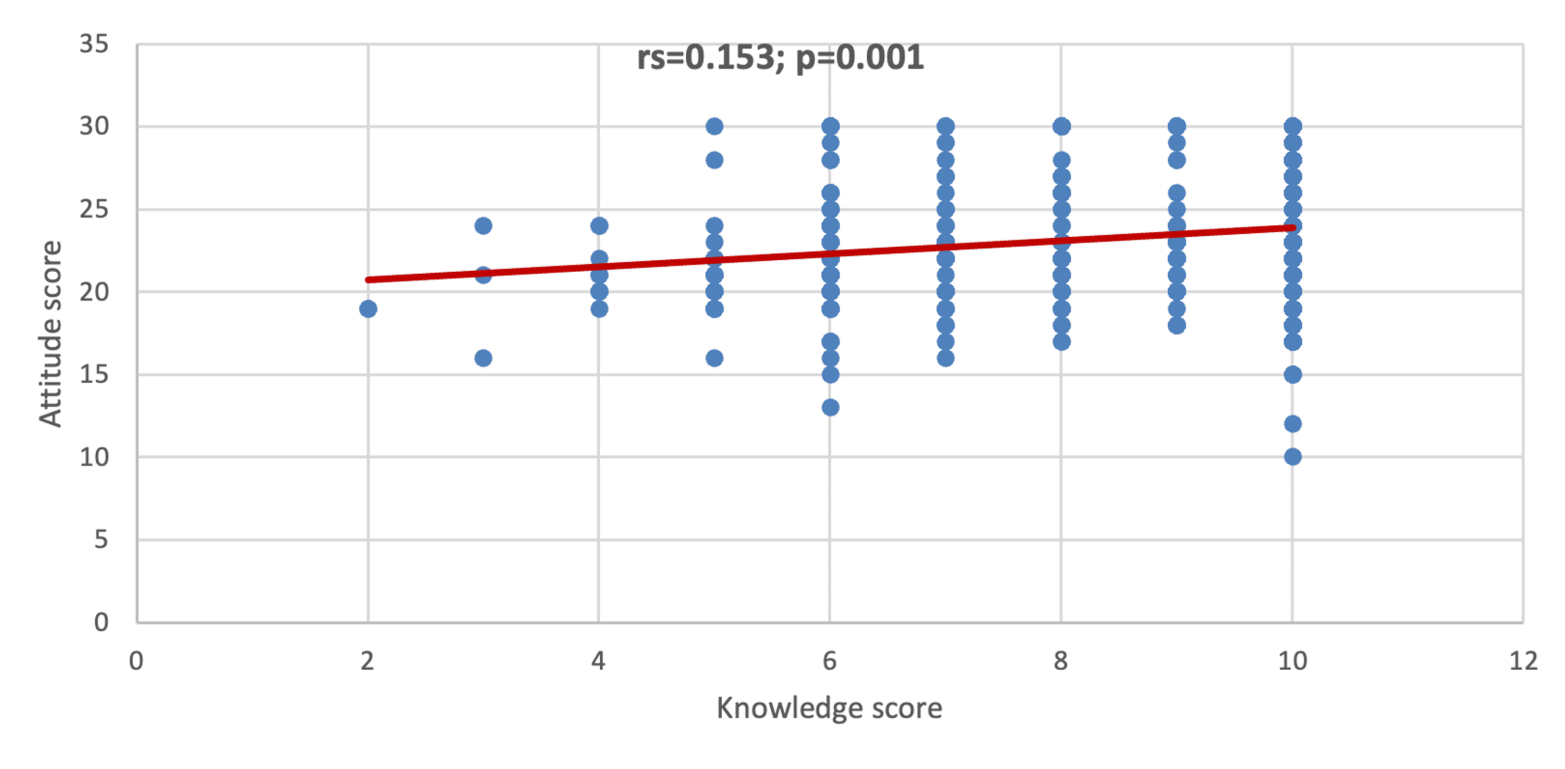
Correlation between knowledge and attitude scores r_s_: Spearman’s rank correlation coefficient

Figure 3 shows a positive significant correlation between the knowledge and attitude scores (Spearman’s rank correlation coefficient, or r_s_=0.153; p=0.001), suggesting that the increase in the knowledge score correlates with the increase in attitude score.

Assessing the differences in knowledge and attitude scores in relation to the socio-demographic characteristics of university students (Table 3), we observed that a higher knowledge score was more associated with increasing age (Z=2.889; p=0.004), increasing academic year level (Z=2.841; p=0.005), living with family (Z=2.760=p=0.006), increasing father's education (Z=2.705; p=0.007), or mother's education (Z=3.594; p<0.001), having sufficient monthly income (Z=2.144; p=0.032), having health insurance (Z=2.717; p=0.007) and use of SM with analgesics (Z=2.851; p=0.004). Regarding attitude, a higher attitude score was more associated with the male gender (Z=5.293; p<0.001), having a pharmacy specialty (Z=2.722; p=0.006), increasing father's education (Z=2.826; p=0.005), or mother's education (Z=4.020; p<0.001), having health insurance (Z=3.020; p=0.003) and the use of SM with analgesics (Z=3.675; p<0.001).

**Table 3:** Differences in knowledge and attitude score in relation to the socio-demographic characteristics of the university students (N=475) ^§ ^P-value has been calculated using Mann Whitney Z-test; ** Significant at p<0.05 level.

Demographic variables	Knowledge score (10) (mean ± SD)	Z-test	P-value^§^	Attitude score (30) (mean ± SD)	Z-test	P-value^§^
Age group						
≤21 years	8.25 ± 1.90	2.889	0.004**	23.5 ± 4.11	1.889	0.059
>21 years	8.75 ± 1.70			22.8 ± 4.01		
Gender						
Male	8.32 ± 1.89	1.093	0.274	24.7 ± 4.58	5.293	<0.001**
Female	8.52 ± 1.81			22.4 ± 3.48		
Marital status						
Single	8.47 ± 1.81	1.234	0.217	23.3 ± 4.08	0.945	0.345
Married	7.74 ± 4.08			22.4 ± 4.26		
Specialty						
Pharmacy	8.37 ± 1.92	0.668	0.504	23.8 ± 4.01	2.722	0.006**
Non-pharmacy	8.51 ± 1.78			22.8 ± 4.09		
Academic year						
Junior students (first-third year)	8.28 ± 1.92	2.841	0.005**	23.3 ± 4.14	0.407	0.684
Senior students (fourth-intern)	8.87 ± 1.55			23.1 ± 3.94		
Living status						
Living with family	8.54 ± 1.79	2.760	0.006**	23.4 ± 4.13	0.760	0.447
Living with dormitory	7.83 ± 2.00			22.5 ± 3.73		
Father education						
Less than university	8.19 ± 1.89	2.705	0.007**	22.5 ± 3.69	2.826	0.005**
University or higher	8.62 ± 1.78			23.8 ± 4.26		
Mother education						
Less than university	8.13 ± 1.93	3.594	<0.001**	22.3 ± 3.67	4.020	<0.001**
University or higher	8.72 ± 1.72			24.0 ± 4.27		
Average monthly income						
Insufficient	8.20 ± 1.77	2.144	0.032**	22.8 ± 3.91	0.708	0.479
Sufficient	8.52 ± 1.86			23.4 ± 4.13		
Health insurance						
No	8.27 ± 1.73	2.717	0.007**	22.4 ± 3.29	3.020	0.003**
Yes	8.57 ± 1.91			23.8 ± 4.49		
Use of self-medication with analgesics						
No	8.12 ± 1.95	2.851	0.004**	22.3 ± 3.78	3.675	<0.001**
Yes	8.65 ± 1.74			23.8 ± 4.17		

## Discussion

This study explores the knowledge and attitude of university students regarding NOAs for SM. The findings of this study will be a great addition to the literature, given the increasing consumption of OTC medication. Moreover, determining students' perspectives on SM would guide university institutions in addressing this growing trend and promoting the safe usage of OTC medications.

Knowledge of non-opioid SM

The knowledge of the students regarding non-opioid SM was deemed sufficient. Based on the given criteria, nearly 60% of the students were considered to have good knowledge, while only fewer than 10% were considered poor (mean score: 8.44 out of 10 points). Further, most students achieved good ratings in all 10 knowledge items, with the highest rating toward the correct definition of analgesic drugs (N=446; 93.9%), while their knowledge was lowest on the type of analgesic drug with a weak anti-inflammatory activity (N=407; 85.7%). Consistent with our findings, several studies documented a good level of understanding of SM among the students [8,21-23]. In contrast, studies conducted among the Indian population [24] and Portuguese students [25] showed poor knowledge of OTC SM. The differences in knowledge may vary according to the region or the number of sample population. However, some other factors could also contribute, including population diversity, education, and other cultural factors.

Significant factor of knowledge

A number of factors contributed to the level of knowledge among university students. In particular, the older age group (>21 years), senior students, those living with family, parents with higher education, having sufficient monthly income, and having insurance were associated with increased knowledge. However, gender, marital status, and specialty showed no correlations with the knowledge score (p>0.05). This contradicted the reports of Alduraibi and Altowayan (2022). Results suggested that female gender and pharmacy students were associated with good knowledge of SMs [8]. Corroborating these reports, Ali et al. (2024) reported that based on the multivariate regression model, females and those working in the medical field were more knowledgeable than their counterparts [24], while in a previous study by Aljadhey et al. (2015), occupation was the only factor associated with the mean knowledge score, which did not align with our results [18]. The variations in findings may be attributed to differences in the study setting, methodology, and sample sizes.

Attitude of non-opioid SM

Despite having good knowledge of non-opioid SM, their attitude about it needs more improvement. The overall mean attitude score was 23.2 out of 30 points, with only 186 (39.2%) being optimistic regarding non-opioid SM. Regarding the specific details of attitude, the highest ratings were seen in attitude items: SM with analgesics may cause interaction with other drugs, can cause side effects, and may have a negative impact. In contrast, the use of analgesics - regardless of the seriousness of the illness - and the sharing of analgesics with relatives and others were identified as the least important items among students. This finding does not align with the study by Alduraibi and Altowayan (2022), which suggested that nearly 60% of students demonstrated high agreement with the attitude assessment of SM [8]. This result is consistent with the report by Ali et al. (2024) [23]. Addressing the gaps in attitudes regarding SM is essential, as these shortcomings may adversely impact health outcomes.

Significant factor of attitude

This study identified various demographic factors influencing attitude. Significant predictors of increased attitude include male gender, pharmacy specialty, parents' higher education, and having health insurance. However, our results indicated that age, marital status, academic year levels, living status, and average monthly income have no relevant effect on attitude. This is almost consistent with the study of Alduraibi and Altowayan (2022). Male students between 21 and 23 years old and pharmacy students were associated with higher attitude scores [8]. Furthermore, we observed a significant positive correlation between knowledge and attitude scores, indicating that an increase in students' knowledge about SM is directly associated with a more positive attitude regarding SM. Further investigation is needed to gain valuable insights that can inform targeted educational interventions aimed at improving health outcomes and addressing the potential adverse effects of SM among students.

Study limitations

Some limitations of the study affect the generalizability of the results. Because the research relied on self-reported online survey data, biases related to recollection and social desirability may have compromised its reliability and accuracy. Additionally, as a cross-sectional survey, it does not measure behavior over time and cannot establish causal relationships between the identified factors.

## Conclusions

The knowledge of university students regarding the SM of NOAs was found to be adequate; however, their attitudes regarding it appear to be lacking. Students with insurance, stable economic backgrounds, and parents who attained higher education were more likely to demonstrate better knowledge and attitudes regarding non-opioid medications. Furthermore, while senior students may possess better knowledge of non-opioid SM, this does not necessarily translate to improved attitudes. Students pursuing a career in pharmacy tended to have more positive attitudes, although their knowledge was not necessarily higher. Additionally, this study provides evidence that the prevalence of SM with analgesics is significant among university students. There is a pressing need to sensitize students about the risks associated with the excessive use of NOAs for SM. Healthcare authorities should intensify efforts to control unsafe medication practices, particularly among students.
